# Military psychological trauma: The contribution of EMDR

**DOI:** 10.1192/j.eurpsy.2023.2078

**Published:** 2023-07-19

**Authors:** D. Falfel, K. Kefi, C. Bechikh, H. Kefi, S. Edhiff, A. Oumaya

**Affiliations:** 1 Psychiatry department, Military Hospital of Tunis, soukra; 2 Psychiatry department, Military Hospital of Tunis, tunis, Tunisia

## Abstract

**Introduction:**

The treatment of psychological trauma is an integral part of the health care provided to military patients in Tunisia. For patients with PTSD several therapeutic options are proposed to patients as EMDR but there are various barriers to the use of EMDR . It seems that being patients consider the therapy which necessitate recognition and expression of emotional distress as a weakness. Added to that patients may be judged negatively by others and experiencing social rejection and isolation which limited the use of EMDR in a military setting.

**Objectives:**

The aim of this study is to study the contribution of the EMDR associated to pharmacological treatment in PTSD among military patients comparing to patients who received only medication .

**Methods:**

The study was conducted since 2021 during ten months at the military hospital concerned two groups of patients with PTSD the first one composed of four military patients with PTSD who received 11 sessions of EMDR associated to medication and the second one four patients exposed to the same trauma under medication only. The evolution oftroubles were assessed using PCL-5 scale and medication compliance was assessed by psychometric scale MARS .Other data was gathered from medical files .

**Results:**

The first group of four military patients who were followed for PTSD since a year under pharmacological treatment presented sleep disorders with flaschbaks and nightmares , . The patients consulted for a depressive disorder and asocial isolation with feelings of insecurity about others. Psychiatrists proposed to associate 11 sessions of EMDR . At the end of sessions the evolution was interesting with regression of symptoms and remission comparing to the second group who presented relapses ten months later.

**Conclusions:**

To improve symptoms of PTSD The use of EMDR is not systematic in a culture where emotional expression is restrictive in an environment that favors the intellect over the emotions. However EMDR can help the military patients with PTSD to link cognition to the emotions. PsychiatristS should propose this therapy as well as possible to patient with severe trauma to avoid relapse .

**Disclosure of Interest:**

None Declared

